# Sensitive immunosensing of melanoma biomarker based on enhanced electrochemiluminescence via electronic metal-support interactions

**DOI:** 10.3389/fchem.2025.1709420

**Published:** 2025-12-12

**Authors:** Jie Pei, Xiaojuan Jia, Fengna Xi, Baolin Zhang

**Affiliations:** 1 Shanxi Bethune Hospital, Third Hospital of Shanxi Medical University, Taiyuan, Shanxi, China; 2 School of Chemistry and Chemical Engineering, Zhejiang Sci-Tech University, Hangzhou, China; 3 Shanxi Medical University, Taiyuan, Shanxi, China

**Keywords:** immunosensor, electrochemiluminescence, layered double hydroxide, electronic met-al-support interaction, melanoma

## Abstract

Developing highly sensitive and convenient immunosensor for the detection of biomarker is important for enhancing the effectiveness of melanoma prevention and control measures. In this work, immunosensor was fabricated for sensitive detection of the melanoma biomarker S100B based on enhanced electrochemiluminescence (ECL) via electronic metal-support interactions. CoAl-layered double hydroxide (LDH) was selected as to modify the costless indium tin oxide (ITO) electrode due to its high surface area and tunable structure. To improve its conductivity and electron transfer capability, oxygen vacancies (Ov) were introduced on LDH through an alkaline etching process, resulting in the LDH-Ov structure. Platinum nanoparticles (Pt) were then *in situ* loaded onto the LDH-Ov surface (Pt@LDH-Ov/ITO). The electronic metal-support interaction (EMSI) between LDH-Ov and Pt nanoparticles played a critical role in improving the catalytic activity, leading to an enhanced ECL signal in the luminol-dissolved oxygen (DO) system. The immunorecognition interface was fabricated on Pt@LDH-Ov/ITO, enabling selective detection of S100B. The constructed immunosensor exhibited a linear detection range for S100B from 100 fg/mL to 100 ng/mL, with a limit of detection (LOD) of 65 fg/mL. The high performance and enhanced sensitivity of the immunosensor make it a promising tool for the early diagnosis, monitoring of recurrence, and personalized treatment of melanoma.

## 1 Introduction

Melanoma is a highly invasive malignant skin cancer with a globally rising incidence, and early diagnosis is critical for improving patient prognosis (Polivka et al., 2024). For early-stage patients, the 5-year survival rate can range from 80% to 100% following surgical excision. However, once the disease progresses to the late stage and metastasis occurs, the 5-year survival rate drops significantly to 10%–25% (Mumford et al., 2018). Early detection plays a key role in enhancing cure rates, preventing tumor metastasis and severe complications, reducing reliance on costly treatments with significant side effects, and easing the physical, psychological, and economic burdens on patients (Harpio and Einarsson, 2004). However, early-stage melanoma often lacks distinct symptoms, making it susceptible to being overlooked or misdiagnosed. There is an urgent need to raise public awareness, promote regular screening, and develop highly sensitive and convenient diagnostic technologies to enable early diagnosis (Posch, 2020), thereby enhancing the effectiveness of melanoma prevention and control measures.

Traditional melanoma diagnosis primarily relies on pathological biopsy as gold standard, which is invasive, difficult to monitor dynamically, and prone to overlooking small metastatic lesions (Posch, 2020). Liquid biopsy, as a non-invasive alternative, provides a promising approach for early screening and disease monitoring of cancers by analyzing circulating biomarkers in blood, such as protein markers and circulating tumor DNA (Bommi et al., 2023; Bommi et al., 2024; Kummari et al., 2023). S100B protein, a member of the S100 calcium-binding protein family, is a homodimer composed of α and β subunits with a molecular weight of approximately 21 kDa. Under normal conditions, S100B is primarily found in glial cells of the central nervous system and melanocytes. However, in melanoma, its expression is significantly elevated and can be secreted into the bloodstream. It has been proven that serum S100B levels are closely correlated with tumor proliferation, invasion, and metastatic potential (Gaynor et al., 1981). Therefore, developing efficient and sensitive methods for S100B detection is crucial for the early diagnosis of melanoma.

Currently, commonly used methods for S100B detection still face challenges in trace-level detection during the early stages. For instance, enzyme-linked immunosorbent assay (ELISA), while offering high specificity, is hindered by time-consuming procedures and limited sensitivity (Wang et al., 2014). Chemiluminescent (CL) immunoassays have improved detection speed but are limited by the poor stability of luminescent reagents, interference from serum matrix components, and high instrument costs (Yuan L. et al., 2024). Electrochemiluminescence (ECL) technology, which applies a voltage to the electrode surface to drive redox reactions in luminescent molecules, generates excited-state intermediates that emit upon returning to the ground state, producing luminescence (Huang J. et al., 2024; Miao, 2008; Wei et al., 2025). ECL offers several advantages, including low background signals, high sensitivity, and a wide linear range (Fan et al., 2024; Ma et al., 2024; Yu et al., 2024; Zhou et al., 2024a). Additionally, it can be easily integrated with immunoassays and nanoparticle modification strategies, significantly enhancing detection performance.

Modifying electrodes with nanomaterials to increase active sites is an effective strategy for enhancing ECL signals (Luo et al., 2022; Shi et al., 2025; Zhang et al., 2024). Layered double hydroxides (LDH), a two-dimensional (2D) layered material, offer significant advantages in electrode modification. Their tunable metal composition and interlayer structure provide a high specific surface area and ordered pores, facilitating the immobilization of luminescent reagents and the loading of nanocatalysts, which improves surface reactivity and mass transfer efficiency (Xian et al., 2023). Additionally, LDH exhibits excellent chemical stability and biocompatibility, making it highly suitable for biosensing applications (Wang K. et al., 2024). The interlayer regions of LDH can host co-reactants or signal molecules to amplify the ECL response (Kim et al., 2021). Moreover, by combining LDH with noble metal nanomaterials to construct an electronic metal-support interaction (EMSI) system, both catalytic activity and signal response can be significantly enhanced. In this system, LDH acts as a two-dimensional support, with its surface rich in hydroxyl groups and a high site density, promoting the uniform anchoring of noble metal nanoparticles. The high specific surface area and ordered pore structure of LDH also facilitate the exposure of more active sites on the noble metal nanomaterials and enhance the mass transfer process. More importantly, the strong EMSI effect between LDH and noble metals can tune the d-band center of the metal, change the adsorption energy of reaction intermediates and greatly enhance the catalytic performance for specific electrochemical reactions (Sang et al., 2023; Xu et al., 2017; Yuan D. et al., 2024). Therefore, constructing an EMSI system based on LDH-noble metal nanomaterials holds great potential for developing high-performance ECL sensing systems.

In this work, CoAl-layered double hydroxide was used to modify the electrode and oxygen vacancies (Ov) were introduced through alkaline etching (LDH-Ov) to enhance its conductivity and electron transfer capability. Platinum nanoparticles (Pt) were then *in situ* loaded onto the LDH-Ov, leveraging the EMSI effect between LDH-Ov and Pt to improve its catalytic activity in the luminol-dissolved oxygen (DO) system, achieving ECL signal enhancement. The high specific surface area and biocompatibility of LDH-Ov enabled efficient immobilization of antibodies, improving sensor specificity and enabling ultra-sensitive detection of S100B. This approach provides a new method for early diagnosis and recurrence monitoring of melanoma.

## 2 Materials and methods

### 2.1 Chemicals and materials

All chemicals used were of analytical grade, and no purification treatment was required prior to use. K_3_[Fe(CN)_6_], K_4_[Fe(CN)_6_], bovine serum albumin (BSA), KCl, potassium hydrogen phthalate (KHP), NaOH, luminol, NaH_2_PO_4_·2H_4_O, Na_2_HPO_4_·12H_2_O, ammonium fluoride (NH_4_F), urea, sodium chloride (NaCl), benzoquinone (BQ), tert-butyl alcohol (TBA), catalase (CAT), Al(NO_3_)_3_·9H_2_O, Co(NO_3_)_2_·6H_2_O, and hexachloroplatinic acid (H_2_PtCl_6_) were purchased from Aladdin Biochemical Technology Co., Ltd. (Shanghai, China). Alpha-fetoprotein (AFP) and interleukin-6 (IL-6) were obtained from Keyu Zhongkai Biotechnology Co., Ltd. (Beijing, China). Interleukin-1β (IL-1β) and placental growth factor (PLGF) were purchased from Shenggong Biotechnology Co., Ltd. (Shanghai, China). C-reactive protein (CRP), neutrophil gelatinase-associated lipocalin (NGAL), S100 calcium-binding protein B (S100B), and anti-S100B monoclonal antibody were purchased from Oukai Biotechnology Co., Ltd. (Nanjing, China). Ultrapure water (18.2 MΩ•cm) was used throughout the experiments. Indium tin oxide (ITO) conductive glass (Kaiwei Optoelectronics Technology Co., Zhuhai, China) with a sheet resistance of <17 Ω/sq and an ITO thickness of 100 ± 20 nm was used. ITO glass was cut into 0.5 cm × 5 cm pieces using a glass cutter, and the electrode area (0.5 cm × 1 cm) was fixed with insulating adhesive. Prior to use, the ITO glass was cleaned in 1 M NaOH solution for 1 h, followed by ultrasonic cleaning in acetone, ethanol, and ultrapure water.

### 2.2 Measurements and instrumentations

The morphology and structure of the functional LDH and the corresponding modified electrodes were characterized using a Zeiss ULTRA 55 scanning electron microscope (SEM). The samples were gold-coated and observed under an acceleration voltage of 5 kV. Elemental and chemical state analyses were performed using a PHI 5300 X-ray photoelectron spectrometer (XPS) with a Mg Kα source (250 W, 15 kV). Cyclic voltammetry (CV) and electrochemical impedance spectroscopy (EIS) were conducted on a Swiss Metrohm Autolab electrochemical workstation (PGSTAT302N) using a three-electrode system: Ag/AgCl as the reference electrode, platinum wire or platinum sheet as the counter electrode, and ITO or modified ITO electrodes as the working electrode. ECL signals were recorded using a Xi’an Ruimei MPI-E instrument. The experiments were carried out in a quartz cell, with luminol (100 μM) as the ECL emitter and 0.01 M PBS (pH = 7.4) as the electrolyte. The potential was scanned in the range of −1.0 to 0.8 V at a scan rate of 100 mV/s. The photomultiplier tube was set to a bias voltage of 700 V, and all tests were performed at room temperature. All ECL experiments in this study were conducted at room temperature (25 °C) and atmospheric pressure.

### 2.3 Modification of LDH on ITO electrode and introduction of oxygen vacancies and platinum nanoparticles

The CoAl-layered double hydroxide was directly grown on ITO using the reported method (Shi et al., 2015). Specifically, 0.33 mM Co(NO_3_)_2_·6H_2_O, 0.11 mM Al(NO_3_)_3_·9H_2_O, 4.0 mM urea, and 1.0 mM NH_4_F were dissolved in 100 mL of ultrapure water with stirring, yielding the precursor solution for LDH growth. The treated ITO electrode was immersed in this solution and reaction was performed at 80 °C for 7 h to obtain the ITO electrode loaded with LDH on its surface (denoted as LDH/ITO). Oxygen vacancies were then introduced on the surface of the LDH/ITO electrode by alkaline etching (Abushrenta et al., 2015; Lu et al., 2012). Specifically, the LDH-Ov/ITO electrode was immersed in a 3 M NaOH solution for 30 min, obtaining the modified LDH-Ov/ITO electrode. Subsequently, platinum nanoparticles were *in situ* electrodeposited on the modified electrode by applying a constant voltage of −0.2 V in a 0.5 mM H_2_PtCl_6_ solution for 2 s, producing the Pt@LDH-Ov/ITO electrode. For comparison, the same procedure was used to electrodeposit Pt nanoparticles on ITO and LDH/ITO electrodes. After deposition, the electrode surface was gently rinsed with ultrapure water.

### 2.4 Preparation of the immunosensor and S100B detection

The Pt@LDH-Ov/ITO electrode was immersed in a solution of S100B antibody (Anti-S100B, 10 μg/mL) and incubated at 4 °C for 60 min. The anti-S100B contains -NH_2_ groups, which interact with Pt-NH_2_ bonds (Li et al., 2021), facilitating the immobilization of the antibody on the surface of the modified electrode, resulting in the Anti-S100B/Pt@LDH-Ov/ITO electrode with an immunorecognition interface. The electrode was then incubated in a solution of bovine serum albumin (BSA, 0.5%, w/w) at 4 °C for 10 min to block non-specific binding sites, yielding the immunosensor (BSA/Anti-S100B/Pt@LDH-Ov/ITO). Throughout the construction of the immunosensor, the electrode was washed with phosphate-buffered saline (PBS, 0.01 M, pH = 7.4) after each modification step to remove any unbound biomolecules from the surface.

For S100B detection, the immunosensor was incubated with varying concentrations of S100B at 4 °C for 60 min. After incubation, the electrode surface was washed with PBS (0.01 M, pH = 7.4) to remove any unbound target molecules. The ECL signal of the electrode was measured before and after S100B binding. The detection solution consisted of PBS (0.01 M, pH = 7.4) containing luminol (100 μM). For the analysis of real samples, the standard addition method was used to determine the S100B content in fetal bovine serum (FBS). The samples were simply diluted 50 times with PBS (0.01 M, pH = 7.4) prior to detection.

## 3 Results and discussion

### 3.1 Construction of LDH-based immunosensor with oxygen vacancies and Pt nanoparticles for ECL signal amplification via EMSI

**FIGURE 1:**
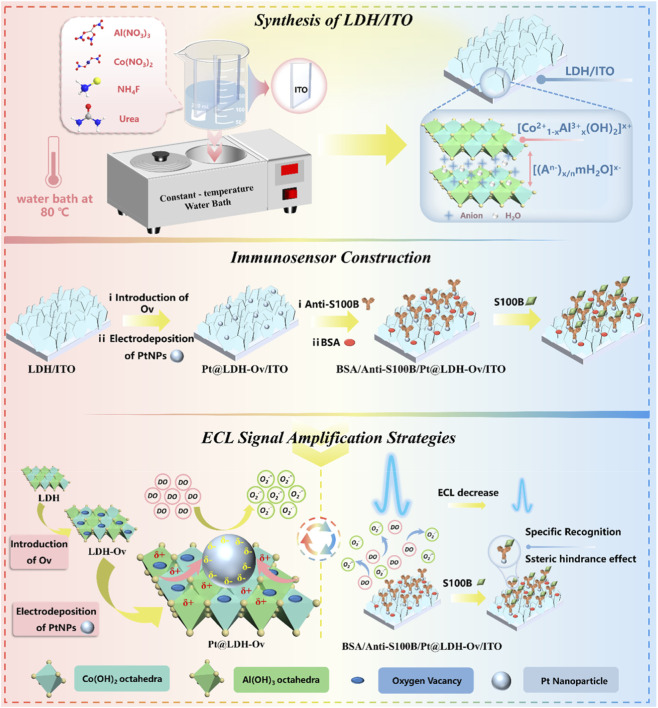
Schematic illustration of LDH preparation, immunosensor construction, and detection of S100B based on EMSI-enhanced ECL.

LDH is hydrotalcite-like material with unique 2D layered structure composed of positively charged metal hydroxide layers and interlayer exchangeable anions. Their general formula is expressed as M^2+^_1-x_M^3+^_x_(OH)_2_A^n-^_x/n_·mH_2_O, where M^2+^ denotes divalent cations (e.g., Ca^2+^, Co^2+^, Fe^2+^, Mg^2+^, Ni^2+^, Zn^2+^), M^3+^ denotes trivalent cations (e.g., Al^3+^, Co^3+^, Cr^3+^, Fe^3+^, Mn^3+^, Ni^3+^), A^n−^ represents interlayer anions (e.g., CO_3_^2-^, SO_4_^2-^, NO_3_^−^, Cl^−^), x is the molar fraction of trivalent cations (M^3+^/(M^2+^ + M^3+^), typically 0.2–0.33), and m is the number of crystallization water molecules (Bi et al., 2022; Wu et al., 2018). In this work, Co^2+^ and Al^3+^ were selected as the divalent and trivalent cations, respectively. CoAl-LDH features excellent biocompatibility and chemical stability. Moreover, alkaline etching introduces abundant oxygen vacancies (Ov) on its surface, which markedly enhance electron transfer and strengthen electronic metal-support interactions (EMSI). Figure 1 presents the preparation of LDH and the construction of immunosensor, which employ EMSI to amplify ECL for S100B detection. During electrode synthesis, Co(NO_3_)_2_ and Al(NO_3_)_3_ served as metal precursors, and the ITO was vertically immersed in the precursor solution. Urea facilitated slow hydrolysis, promoting two-dimensional crystallization and the formation of stable ultrathin nanosheets. NH_4_F acted as a mineralizer and morphology-directing agent, inducing heterogeneous nucleation and oriented growth of LDH nanocrystals perpendicular to the substrate, ultimately generating nanosheet wall-like architectures (Shi et al., 2015).

For immunosensor construction, the LDH/ITO electrode was etched under alkaline conditions to introduce abundant oxygen vacancies (LDH-Ov/ITO), followed by electrodeposition of platinum nanoparticles (Pt). The amino groups (-NH_2_) of S100B antibodies coordinated with Pt through Pt-NH_2_ interactions (Li et al., 2021), enabling their stable immobilization on the electrode surface and yielding the Anti-S100B/Pt@LDH-Ov/ITO interface. Subsequently, the electrode was incubated in BSA to block nonspecific adsorption, affording the final immunosensor (BSA/Anti-S100B/Pt@LDH-Ov/ITO). The strong EMSI between PtNPs and LDH-Ov induced interfacial charge redistribution, enriching the electron density on Pt (Pt^δ−^) and accelerating electron transfer between Pt and LDH-Ov. This synergistic effect facilitated electron transport from the support to ECL reactants (e.g., O_2_^·-^), reduced the reaction energy barrier, and thereby enhanced the ECL response. In addition, Pt^δ−^-Ov-Co^3+^ interfacial sites promoted efficient adsorption of DO, generating higher concentrations of superoxide radicals and further amplifying ECL emission. In presence of S100B, antigen-antibody binding occurred at the sensing interface, forming immunocomplexes that increased interfacial resistance and steric hindrance, thereby restricting diffusion of ECL emitters and decreasing the ECL signal. These signal change enabled sensitive detection of S100B.

### 3.2 Characterization of functionalized LDH materials and modified electrodes

**FIGURE 2:**
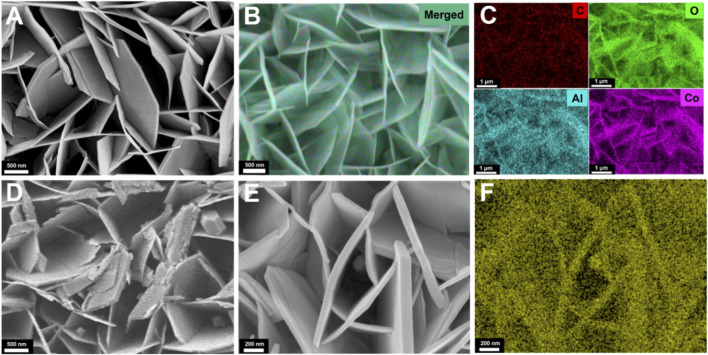
**(A)** SEM image of LDH/ITO. **(B,C)** EDS elemental mapping images (merged and C, Co, Al, and O) of for LDH/ITO. **(D)** SEM image of LDH-Ov/ITO. **(E)** SEM image of Pt@LDH-Ov/ITO. **(F)** EDS Pt elemental mapping of Pt@LDH-Ov/ITO.

The morphology of LDH, LDH-Ov, and Pt@LDH-Ov/ITO was characterized by SEM. As shown in Figure 2A, large, smooth, and flat 2D nanosheets of LDH were vertically grown on the ITO substrate. Energy-dispersive X-ray spectroscopy (EDS) elemental mapping (Figures 2B,C) revealed the presence of C, O, Co, and Al, confirming the successful deposition of LDH on ITO. After sodium hydroxide etching, the SEM image of LDH (Figure 2D) shows cracks and collapse in the nanosheet structure, likely due to the leaching of Al during the alkaline treatment (Zhou et al., 2020). Following the deposition of platinum nanoparticles onto the LDH-Ov surface, uniform nanoparticles were observed (Figure 2E), and EDS mapping confirmed that these particles consist of platinum (Figure 2F), indicating the successful loading of platinum nanoparticles onto the LDH-Ov/ITO electrode.

Cyclic voltammetry (CV) was performed to examine the characteristic peaks of Pt. As shown in Supplementary Figure S1 (Supplementary Material), two pairs of reduction peaks (c1, c2) and oxidation peaks (a1, a2) were observed on the Pt@LDH-Ov/ITO electrode. The reduction peak (c1) and oxidation peak (a1), located in the negative potential range, correspond to the adsorption and desorption of hydrogen atoms on the platinum surface. The additional broad peaks (a2, c2) in the positive potential range correspond to the formation and reduction of platinum oxides, further confirming the successful deposition of platinum nanoparticles (Chen et al., 2011).

**FIGURE 3:**
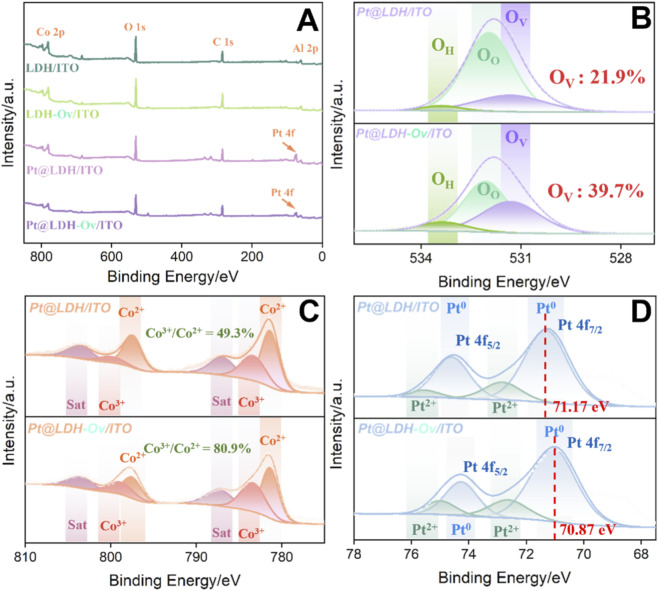
**(A)** XPS survey of LDH/ITO, LDH-Ov/ITO, Pt@LDH/ITO and Pt@LDH-Ov/ITO. **(B–D)** High-resolution XPS of O 1s **(B)**, Co 2p **(C)** and Pt 4f **(D)** of Pt@LDH/ITO and Pt@LDH-Ov/ITO.

To investigate the distribution of structural elements and Pt deposition, X-ray photoelectron spectroscopy (XPS) analysis was conducted on Pt@LDH-Ov/ITO. Figure 3A presents the full XPS spectra of LDH/ITO, LDH-Ov/ITO, Pt@LDH/ITO, and Pt@LDH-Ov/ITO. Characteristic peaks for C 1s, O 1s, Co 2p, and Al 2p were observed in all samples (Huang H. et al., 2024), validating the presence of LDH on the electrode surface. After Pt deposition, a Pt 4f peak appeared at the corresponding binding energy, confirming the successful loading of platinum nanoparticles. To explore the EMSI between Pt and LDH-Ov, high-resolution XPS analysis was performed. As shown in the O 1s spectrum in Figure 3B, three peaks were observed at 532.6 eV, 531.9 eV, and 531.2 eV for both Pt@LDH/ITO and Pt@LDH-Ov/ITO electrodes. These peaks correspond to surface-adsorbed water (O_H_), oxygen adsorption in the layered double hydroxides (Oo), and low-coordination oxygen vacancies (Ov), respectively (Wu et al., 2026; Zeng et al., 2020). After alkaline etching, the peak corresponding to oxygen vacancies increased from 21.9% to 39.7%, confirming the successful introduction of additional oxygen vacancies. The Co 2p spectrum (Figure 3C) shows characteristic peaks for Co^2+^ at 781.3 eV and 797.2 eV, and for Co^3+^ at 783.2 eV and 798.7 eV, with satellite peaks observed at 786.6 eV and 802.9 eV (Jing et al., 2019; Zhu et al., 2025a). The coexistence of Co^2+^ and Co^3+^ indicates a valence change in cobalt. Notably, the Co^3+^/Co^2+^ peak area ratio increased from 49.3% to 80.9%, which is essential for the EMSI effect and suggests the formation of Ov-Co^3+^ active sites. In the Pt 4f spectra (Figure 3D), the Pt^0^ 4f peak in Pt@LDH/ITO was observed at 71.17 eV, while in Pt@LDH-Ov/ITO, this peak shifted to a lower binding energy of 70.87 eV. This negative shift in binding energy suggests an increased electron density on the Pt species, forming an electron-rich Pt (Pt^δ−^) state (Yuan D. et al., 2024; Zhang et al., 2023). This result strongly supports the occurrence of the EMSI effect at the Pt@LDH-Ov interface, where electrons transfer from the LDH-Ov support to Pt, forming an active interface structure with Pt^δ−^-Ov-Co^3+^.

### 3.3 Mechanism of EMSI-induced enhancement of ECL signal of luminol-DO

**FIGURE 4:**
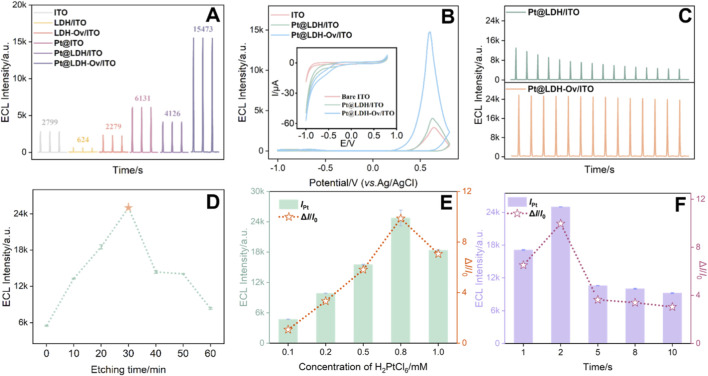
**(A)** ECL responses obtained on different electrodes. **(B)** ECL-voltage curves obtained on different electrodes. The inset shows the corresponding CV curves. **(C)** ECL-time curves for Pt@LDH/ITO and Pt@LDH-Ov/ITO electrodes. **(D–F)** ECL response obtained on Pt@LDH-Ov/ITO fabricated under different etching time for introducing oxygen vacancies **(D)**, precursor concentration **(E)** and deposition time **(F)** for Pt deposition.

Figures 4A,B compared the ECL signals of different electrodes. The LDH modified electrode (LDH/ITO) showed negligible ECL signals, which was attributed to the poor conductivity of LDH itself. The LDH-Ov modified electrode exhibited a 2.8-fold enhancement in ECL intensity relative to the LDH modified electrode, suggesting that oxygen vacancies contribute significantly to the signal amplification. In addition, electrochemical impedance spectroscopy (EIS) revealed a decrease in the charge transfer resistance (*R*ct) for the LDH-Ov electrode compared to the LDH electrode (from 45 Ω to 38Ω), indicating improved electron transfer (Supplementary Figure S2). After the deposition of platinum nanoparticles, the ECL signal of the Pt@LDH/ITO electrode increased, confirming that Pt can catalyze the enhancement of the ECL signal. In contrast, the ECL signal drastically increased when PtNPs were deposited onto LDH-Ov, which was rich in oxygen vacancies. This demonstrated that the EMSI effect between LDH-Ov and PtNPs significantly enhanced the ECL response. In addition, the ECL signal and the stability measured on the Pt@LDH-Ov/ITO electrode was significantly superior to that on the Pt@LDH/ITO electrode (Figure 4C). The Pt@LDH-Ov/ITO electrode exhibited high stability over 14 consecutive scans, as evidenced by a relative standard deviation (RSD) of 2.7% in ECL signal. The reproducibility of the electrode, as well as the stability in repeated measurement and storage were also investigated. Five independently prepared Pt@LDH-Ov/ITO electrodes fabricated in parallel under the same conditions exhibited an RSD of only 0.7% in ECL response, demonstrating excellent reproducibility in the electrode preparation process (Supplementary Figure S3). When measured repeatedly five times under identical conditions, the Pt@LDH-Ov/ITO electrode showed no significant signal change, with RSD of approximately 2.8% (Supplementary Figure S3). The electrode also maintained strong ECL signal over 3 days of storage with only a signal decrease of 5.0% (Supplementary Figure S4), confirming its stability under both repeated use and storage conditions.

The replacement of metal elements was also performed. When the metal was changed to Pd, the electrode deposited with Pd exhibited unstable ECL signals (Supplementary Figure S5). This instability might be attributed to the chemical inertness of Pd. The standard reduction potential of Pd (approximately 0.987 V vs. SHE) is significantly lower than that of Pt (1.18 V vs. SHE), making Pt relatively more reactive. For instance, Pd surfaces are prone to the formation of oxide layers such as PdO and Pd(OH)_2_. These substances can reduce electron transfer efficiency at the electrode surface and hinder the electron transfer between ECL intermediates and the electrode. Furthermore, partial dissolution or detachment of these oxide layers during ECL reactions may lead to instability in the number and distribution of active sites, thereby causing unstable ECL signal. In the case of Ag, it was observed that Ag undergoes electrochemical dissolution at around 0.35 V. In this work, besides serving as catalysts, the metal nanoparticles are also required to act as carriers for antibody immobilization to construct the immunosensor. The dissolution of Ag would compromise the stability of the immobilized antibody. Thus, Pt was selected for further investigation.

To further optimize the fabrication conditions for Pt@LDH-Ov/ITO electrode, the etching time of LDH/ITO for introducing oxygen vacancies, precursor concentration and deposition time for Pt deposition were systematically optimized. As shown in Figures 4D–F, the strongest and most stable ECL signal was observed when the etching time for introducing oxygen vacancies was 30 min, precursor concentration was 0.8 mM, and deposition time was 2 s. These conditions were chosen for further investigation.

**FIGURE 5:**
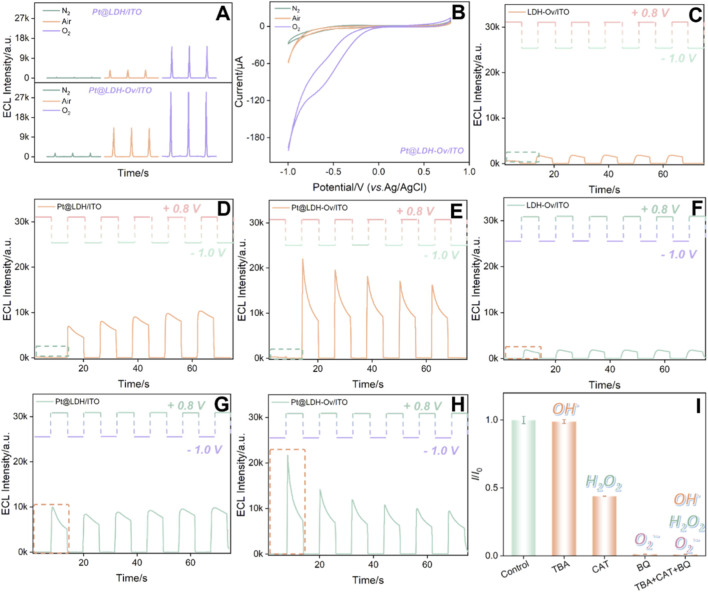
ECL **(A)** and CV **(B)** curves obtained on different electrodes under varying atmospheres. **(C–H)** ECL responses of different electrodes at different initial step potentials (**C–E**, +0.8V, **F–H**, −1.0V). **(I)** *I*/*I*_0_ of Pt@LDH-Ov/ITO in PBS (0.01 M, pH = 7.4) containing luminol (100 μM), with the addition of BQ (100 μM), TBA (100 μM) and CAT (100 μM), respectively, where *I* and *I*_0_ are ECL signals in the presence or absence of ROS radical scavenger, respectively. The schematic diagram above the ECL curve in **(C**–**H)** shows the corresponding voltage in the measurement.

To explore the mechanism behind the high ECL signal from Pt@LDH-Ov/ITO, CV and ECL tests were conducted under different atmospheric conditions. Figures 5A,B show the ECL and CV curves for Pt@LDH/ITO and Pt@LDH-Ov/ITO electrodes under varying atmospheres. The results indicate that the reduction current was highest and the ECL signal strongest in an oxygen atmosphere, suggesting that reactive oxygen species (ROS) generated from DO act as co-reactant in the reaction (Zhao et al., 2025; Zhou et al., 2025). In addition, the ECL signal and CV current for Pt@LDH-Ov/ITO were significantly higher than those for Pt@LDH/ITO, further confirming that the EMSI effect enhances the catalytic activity of Pt, promoting more ROS generation and thereby significantly amplifying the signal.

To investigate the mechanism of Pt in this ECL system, step-pulse tests were conducted to analyze the reaction process. Figures 5C–H showed the ECL responses of LDH/ITO, Pt@LDH/ITO, LDH-Ov/ITO, Pt@LDH/ITO, and Pt@LDH-Ov/ITO electrodes at different initial step potentials. When the initial potential was set to 0.8 V (Figures 5D–F), luminol will be electrochemically oxidated. Specifically, the luminol anion (LH^−^) was electrooxidized to generate free radicals (L·^-^). In the absence of co-reactants, the L·^-^ free radicals exhibited low luminescence efficiency. When the potential stepped to −1.0 V, an oxygen reduction reaction (ORR) occurred, generating ROS. However, L·^-^ had a short lifetime and could not react with ROS in time, resulting in no significant ECL signal. Upon stepping the potential back to 0.8 V, long-lived ROS accumulated from the previous step, reacting with the newly generated L·^-^ to produce ECL (Fan et al., 2025a; Fan et al., 2025b). Similarly, when the initial potential was set to −1.0 V and then stepped to 0.8 V (Figures 5G,H), a clear transition from no signal to a detectable ECL signal was observed. Notably, the Pt@LDH-Ov/ITO electrode exhibited much higher ECL signals at the initial potential of −1.0 V than the other electrodes, demonstrating that Pt significantly catalyze the electroreduction of dissolved oxygen. Moreover, Pt@LDH-Ov/ITO showed catalytic activity for the electrooxidation of luminol. To further identify the specific ROS species involved in the reaction, ROS scavenging experiments were performed (Figure 5I). The results showed that the addition of quinone, a scavenger for O_2_^·-^, nearly completely quenched the ECL signal, indicating that O_2_^·-^ is the primary ROS involved in the luminol luminescence process (An et al., 2024; Zhou et al., 2024b).

Thus, the ECL mechanism based on the EMSI-enhanced luminol-DO system can be summarized as the following Equations 1–5:

$$O_{2}+e^{-}\overset{\text{PtNPs}}{\to }O_{2}^{\cdot -}$$ (1)

$$O_{2}^{\cdot -}+2H_{2}O+e^{-}\to H_{2}O_{2}+2OH^{-}$$ (2)

$${\text{LH}}^{-}-e^{-}\to L^{\cdot -}+H^{+}$$ (3)

$$L^{\cdot -}+O_{2}^{\cdot -}\to {\text{AP}}^{2-*}+N_{2}$$ (4)

$${\text{AP}}^{2-*}\to {\text{AP}}^{2-}+hv$$ (5)

### 3.4 Feasibility and optimization of construction conditions for the immunosensor

**FIGURE 6:**
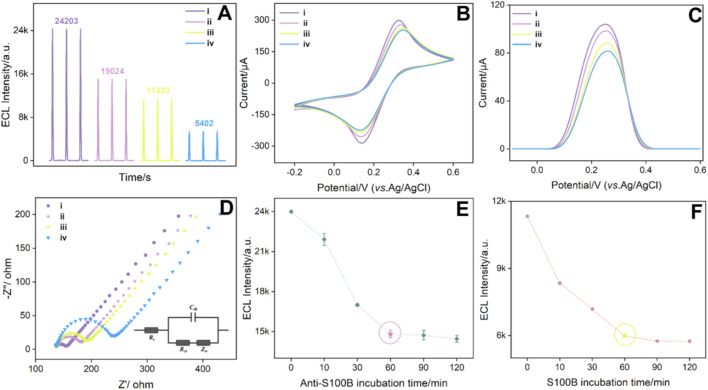
ECL signals of different electrodes **(A)**, CV curves **(B)**, DPV curves **(C)**, and EIS curves **(D)**. The inset in **(D)** was the corresponding equivalent circuit*.* The S100B antigen concentration was 1 ng/mL. ECL measurements were performed in PBS (0.01 M, pH = 7.4) containing 100 μM luminol. The measurements in **(B–D)** were conducted in a solution of 0.1 M KCl containing 2.5 mM Fe(CN)_6_^3-/4-^. ECL signals of the electrodes during the construction of the immunosensor at different S100B antibody incubation times **(E)** and during S100B incubation with the immunorecognition interface **(F)**. The cycles in **(D)** and **(E)** indicated the chosen condition for further experiments. The error bars were estimated using three parallel measurements.

An immunosensor was fabricated by constructing the immunorecognition interface on the surface of the Pt@LDH-Ov/ITO electrode. The feasibility of immunosensor fabrication was investigated by measuring changes in the ECL signal during the construction process (Figure 6A). As the electrode underwent stepwise modification, the ECL intensity progressively decreased. To evaluate the interfacial properties of the electrode after each modification step, CV, differential pulse voltammetry (DPV), and electrochemical impedance spectroscopy (EIS) were employed, using potassium ferrocyanide/ferrocyanide as the electrochemical probe. Figure 6B shows the CV curves during the modification process. After platinum deposition onto the LDH-Ov surface, a pair of reversible redox peaks appeared, indicating that the composite structure facilitates electron transfer. Subsequent bio-modification with antibodies and BSA blocking layers resulted in a gradual decrease in the CV peak currents, primarily due to the insulating nature of the antibody and BSA layers (Guo et al., 2023; Yan et al., 2025; Zhou et al., 2023; Zhu et al., 2025b). These non-conductive biomolecular layers not only increased electron transfer resistance but also introduced steric hindrance, limiting the diffusion of probe molecules to the electrode surface (Chen et al., 2022; Gong et al., 2022a; Gong et al., 2022b; Huang et al., 2023). As a result, the DPV current response weakened (Figure 6C), that was consistent with the variation trend of the CV peak current. The *R*ct from EIS was employed to further verify the changes in the interfacial resistance of the electrode during the construction of the immunosensor (Figure 6D). After modifying the Pt@LDH-Ov/ITO electrode (*R*ct 22 Ω) with the detection antibody, the *R*ct of the Anti-S100B/Pt@LDH-Ov/ITO electrode increased (49 Ω). The *R*ct further increased (60 Ω) for the immunosensor obtained after blocking with BSA. This was attributed to the introduction of non-conductive biomolecules on the electrode surface, which increased the interfacial resistance. When S100B was present, the Anti-S100B antibody at the recognition interface specifically bound to S100B, forming an immunocomplex. This binding resulted in a more pronounced steric hindrance effect of the biomolecules at the interface, leading to a decrease in the ECL signal, further reduction in the CV and DPV current responses, and a continued increase in electrode *R*ct (103 Ω). Based on these observations, a signal-off sensing mechanism was proposed, where the interfacial mass transfer efficiency gradually decreases, enabling highly specific detection of the target molecule.

To optimize the detection performance, the incubation time of the S100B antibody during the construction of the immunorecognition interface (Figure 6E) and the incubation time during S100B detection (Figure 6F) were systematically evaluated. The results indicated that the ECL signal stabilized after a 60 min incubation of the S100B antibody during the construction of the immuno-recognition interface. Similarly, when the S100B detection incubation time was 60 min, the ECL signal also reached a stable value. Further extension of the incubation time did not result in any significant decrease in the ECL signal, indicating that the antibody-S100B binding had reached stability. Thus, these conditions were chosen for further investigation.

### 3.5 Performance for the detection of S100B

**FIGURE 7:**
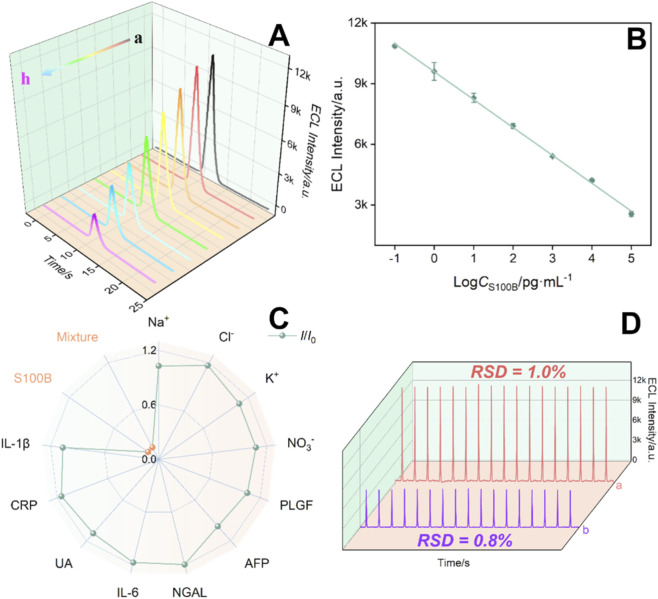
**(A)** ECL signals of the immunosensor after incubation with different concentrations of S100B. **(B)** Linear regression curve of ECL intensity versus the logarithmic value of S100B concentration. The error bars were estimated using three parallel measurements. **(C)** ECL signals of the immunosensor after incubation with different substances. **(D)** Continuous ECL measurements of the immunosensor before (upper line) and after (bottom line) incubation with S100B.

Under optimal experimental conditions, the fabricated immunosensor, BSA/Anti-S100B/Pt@LDH-Ov/ITO electrode, was incubated with S100B solutions of varying concentrations, followed by ECL signal measurement to evaluate its detection performance. The resulting ECL curves were shown in Figure 7A. As the S100B concentration increased, the ECL signal of the immunosensor gradually decreased. In the concentration range from 100 fg/mL to 100 ng/mL, a good linear relationship was observed between the ECL intensity (*I*_ECL_) and the logarithmic concentration of S100B (log*C*_S100B_). The linear regression equation was *I*_ECL_ = −1,379 (±22) log*C*_S100B_ (pg/mL) + 9,594 (±62) with a regression coefficient of 0.998 (Figure 7B). The detection limit (LOD) was calculated to be 65 fg/mL. Comparison of the detection performance of S100B by different analytical methods was shown in Supplementary Table S1 (Chen et al., 2024; Liu et al., 2013; Rodríguez et al., 2021; Wang et al., 2014; Wang TT. et al., 2024; Yuan L. et al., 2024). The LOD obtained with the developed method was lower than immunoassays based on EIS detection using BSA/Anti-S100B/cysteamine/gold electrode (BSA/Anti-S100B/Cys/AuE), or resonance rayleigh scattering (RRS) detection using BSA/Anti-S100B/Cys-gold nanoparticles (BSA/Anti-S100B/Cys-AuNPs) probe, or surface-enhanced Raman scattering (SERS) detection based on sandwich immunosensing using molybdenum trioxide-copper(Ⅲ) sulfide-chitosan-detection antibody/S100B/capture antibody (MoO_3-X_-CuS(iii)-CS-dAb/S100B/cAb), or differential pulse voltammetry (DPV) detection based on sandwich immunosensor using alkaline phosphatase-dAb/S100B/cAb/glutaraldehyde/polyethylenimine/polymethyl methacrylate microfluidic chip (ALP-dAb/S100B/cAb/GA/PEI/PMMAMC). The LOD obtained with the present method was also lower than peptide-based square wave voltammetry (SWV) sensing using C_16_ alkyl chain-signal peptide-Fc/S100B/6-mercapto-1-hexanol/cysteine-4 proline-capture peptide/AuE (C_16_-Signal Pep-Fc/S100B/MCH/CP_4_-Capture Pep/AuE) or Cu-Signal Pep/S100B/9-mercapto-1-nonanol/Capture Pep/AuE (Cu-Signal Pep/S100B/MN/Capture Pep/AuE). The ECL immunosensor constructed herein exhibits dual advantages of operational simplicity and high detection sensitivity. Firstly, the operational convenience is achieved by simplifying the assay protocol, which requires only one recognition antibody and eliminates cumbersome enzymatic labeling steps. Secondly, the enhanced sensitivity is attributed to the combination of the inherently low background of ECL and signal amplification via the EMSI effect, which collectively yield a high signal-to-noise ratio. This stands in clear contrast to conventional ELISA or magnetic bead-based sandwich immunoassay, where the multi-step sandwich formation, antibody labeling often lead to complicated procedures and limited sensitivity.

The selectivity and anti-interference capability of the fabricated ECL immunosensor were evaluated by testing its response to various potential interferents commonly found in biological samples (Figure 7C). These included inorganic ions (Na^+^, Cl^−^, K^+^, NO_3_^−^), the redox-active metabolite uric acid (UA), cytokines such as interleukin-6 (IL-6) and interleukin-1β (IL-1β), placental growth factor (PLGF), as well as tumor biomarkers including alpha-fetoprotein (AFP), C-reactive protein (CRP), and neutrophil gelatinase-associated lipocalin (NGAL), both individually and in mixture. As shown in Figure 7C, the ECL signal showed no significant change upon introduction of these interfering substances. A notable change in the ECL signal was observed only when the sensor was incubated with the target protein S100B or the mixture containing S100B. These results confirm the high specificity of the immunosensor toward S100B, demonstrating good selectivity and anti-interference performance.

In addition, continuous ECL scanning for 17 cycles was performed on the electrode before and after S100B incubation. As shown in Figure 7D, the signals remained stable for both cases, with RSD of 1.0% and 0.8%, respectively, indicating that the sensor exhibits high stability during the detection process. Additionally, the ECL signals of five parallel sensors after antigen incubation were analyzed. The ECL signals displayed excellent consistency, with an RSD value of 0.9%, confirming the high reproducibility of the fabricated immunosensor.

### 3.6 Real sample analysis

To evaluate the practical applicability of the constructed sensor for S100B, spiked serum samples were analyzed using the standard addition method to assess the accuracy and precision of the sensor for detecting S100B. As shown in Table 1, the spiked recovery rate of S100B in serum samples ranged from 95.1% to 98.0%, with the RSD of three parallel measurements ranging from 0.5% to 3.4%. These results demonstrate high recovery rates and excellent detection reproducibility.

**TABLE 1:** ECL detection of S100B using the fabricated immunosensor.

Sample	Added^a^ (pg/mL)	Found (pg/mL)	RSD (%, n = 3)	Recovery (%)
Fetal bovine serum^a^	1.00	0.951	3.4	95.1
	10.0	96.5	4.0	96.5
	1.00 × 10^4^	9.80 × 10^3^	0.5	98.0

^a^ The fetal bovine serum was diluted by 50 times using PBS (0.01 M, pH 7.4) before detection.

## 4 Conclusion

In this work, platinum nanoparticles were modified onto the surface of the LDH-Ov/ITO electrode via an electrodeposition method. Based on the EMSI effect, this modification significantly enhanced the ECL signal of the luminol-DO system, enabling highly sensitive detection of S100B protein. The reaction mechanism was further investigated. The results indicated that platinum nanoparticles deposited on the LDH surface exhibited excellent catalytic performance, effectively promoting the generation of ROS, which in turn greatly amplified the ECL signal of the luminol-DO system. Based on this, an immunosensor was successfully constructed, achieving sensitive detection of S100B with low limit of detection. This work significantly enhances the ECL performance of the luminol-DO system and holds promise for expanding applications in melanoma biomarker detection, offering new approaches for disease diagnosis and health monitoring. In this work, ITO was chosen as the electrode material due to its low cost and excellent optical transparency despite its inherent rigidity. Future research could focus on utilizing flexible electrodes, which would pave the way for wearable sensors. Integration with wireless communication technologies, such as Bluetooth, would enable real-time data transmission and early warning alerts. In addition, combining ECL immunosensors with microfluidic technology could lead to highly integrated lab-on-a-chip systems, offering potential for enhanced automation and a significant reduction in sample and reagent consumption.

## Data Availability

The original contributions presented in the study are included in the article/Supplementary Material, further inquiries can be directed to the corresponding authors.
