# Supplementary material for: Sensitive immunosensing of melanoma biomarker based on enhanced electrochemiluminescence via electronic metal-support interactions
Source: Front Chem. 2025 Dec 12;13:1709420. doi: 10.3389/fchem.2025.1709420 (PMC12740906; doi:10.3389/fchem.2025.1709420)
Supplement: Supplementary file 1 [file DataSheet1.docx]

**TABLE**

Table S1 Comparison of the detection performance of S100B by different analytical methods.

| Sensing platform | Method | Linear range  (pg/mL) | LOD  (pg/mL) | Ref. |
| --- | --- | --- | --- | --- |
| BSA/Anti-S100B/Cys/AuE | EIS | 10 ~ 1.0×10^3^ | 6.0 | [56] |
| BSA/Anti-S100B/Cys-AuNPs | RRS | 50 ~ 4.5×10^3^ | 2.0 | [57] |
| C_16_-Signal Pep-Fc/S100B/MCH/CP_4_- Capture Pep/AuE | SWV | 2.1 ~ 1.3×10^2^ | 0.21 | [58] |
| Cu-Signal Pep/S100B/MN/Capture Pep /AuE | SWV | 2.1 ~ 5.4×10^2^ | 2.1 | [9] |
| MoO_3-X_-CuS₍ᵢᵢᵢ₎-CS-dAb/S100B/cAb | SERS | 1.0 ~ 1.0×10^5^ | 0.47 | [59] |
| ALP-dAb/S100B/cAb/GA/PEI/PMMAMC | DPV | 0.10 ~ 1.0×10^2^ | 0.10 | [60] |
| BSA/Anti-S100B/Pt@LDH-Ov/ITO | ECL | 0.10 ~ 1.0×10^5^ | 0.065 | This work |

Cys, cysteamine; AuE, gold electrode; EIS, Electrochemical Impedance Spectroscopy; AuNPs, gold nanoparticles; RRS, resonance rayleigh scattering; C_16_, C_16_ alkyl chain; Pep, peptide; MCH, 6-mercapto-1-hexanol; CP_4_, cysteine-4 proline; SWV, square wave voltammetry; MN, 9-mercapto-1-nonanol; MoO_3-X_-CuS₍ᵢᵢᵢ₎-CS-dAb, molybdenum trioxide-copper(Ⅲ) sulfide-chitosan-detection antibody; cAb, capture antibody; SERS, surface-enhanced Raman scattering; ALP, alkaline phosphatase; GA, glutaraldehyde; PEI, polyethylenimine; PMMAMC, polymethyl methacrylate microfluidic chip; DPV, differential pulse voltammetry.

**FIGURES**


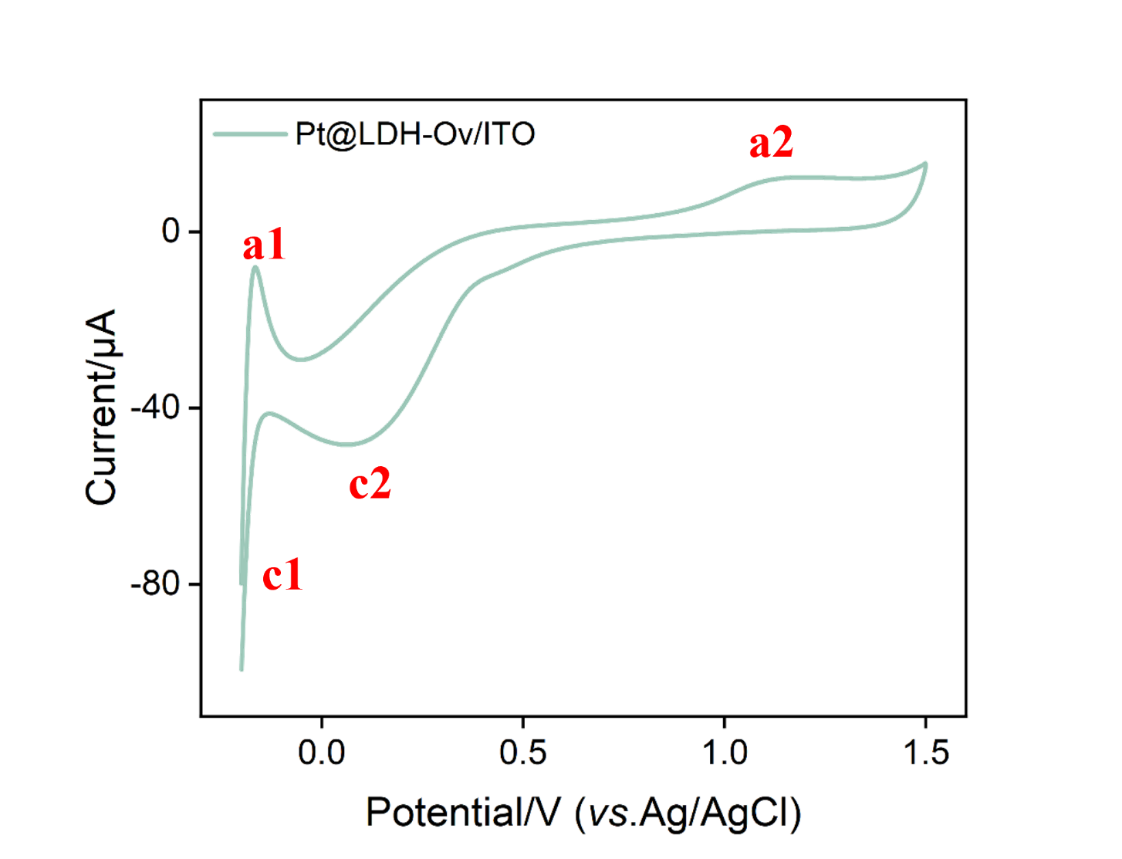


Figure S1 CV curves of Pt@LDH-Ov/ITO in 1 M H_2_SO_4_. The scan rate is 100 mV/s.


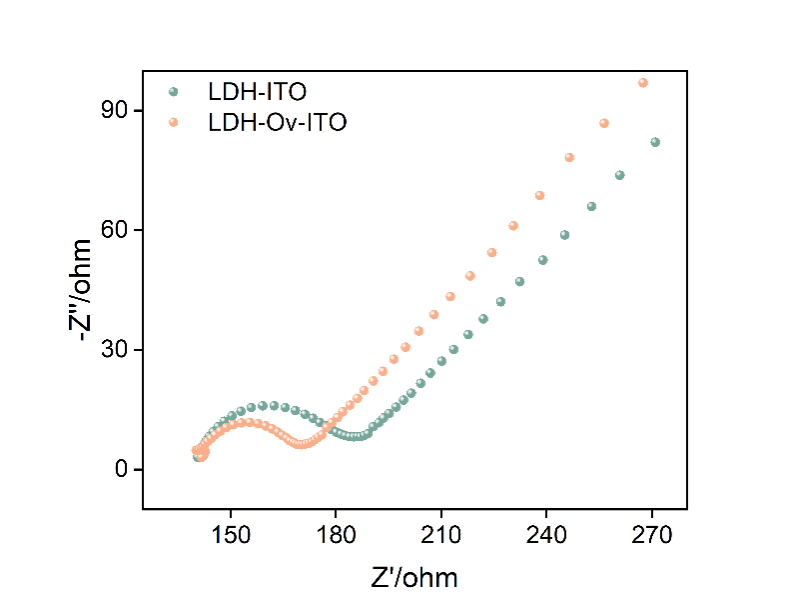


Figure S2 EIS curves of different electrodes in 0.1M KCl containing 2.5 mM [Fe(CN)_6_] ^3-/4-^. The CV scan rate is 50 mV/s.


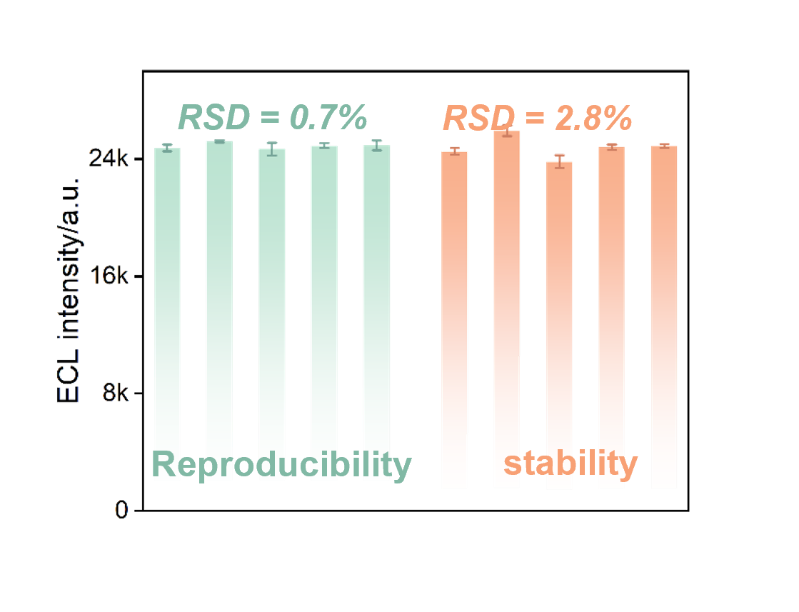


Figure S3 The signal stability in repeat measurements and reproducibility of Pt@LDH-Ov/ITO. The scan rate is 100 mV/s.


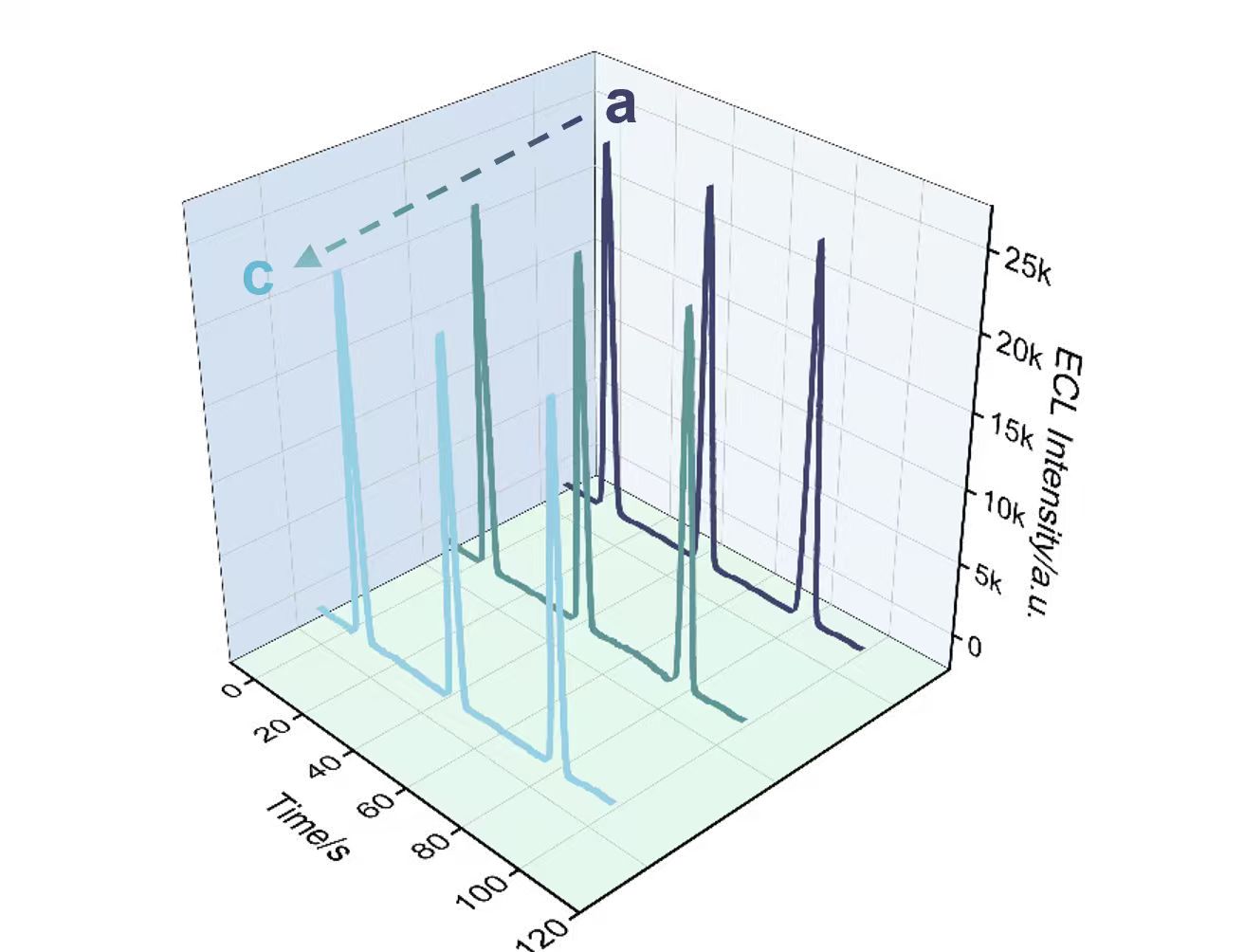


Figure S4 ECL signal obtained on Pt@LDH-Ov/ITO in different storage time (a-c 1-3 days).


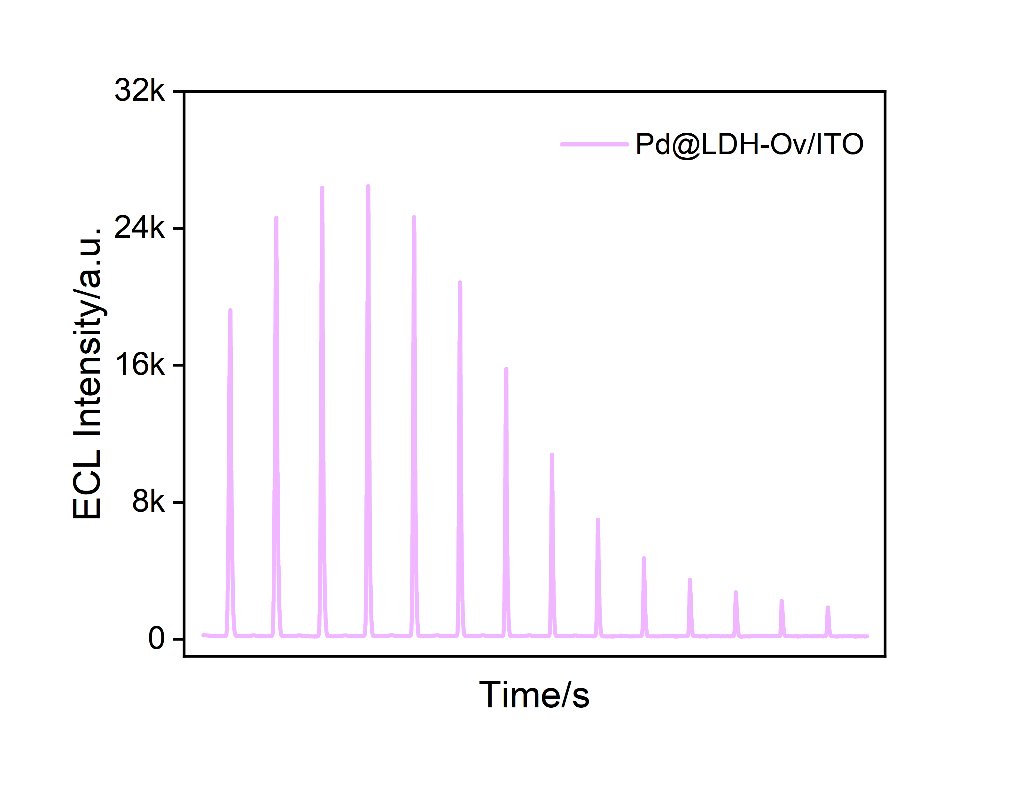


Figure S5 ECL curve obtained on Pd@LDH-Ov/ITO electrode.
